# Scopolamine infusion in the basolateral amygdala after saccharin intake induces conditioned taste avoidance in rats

**DOI:** 10.1007/s00213-024-06624-7

**Published:** 2024-06-01

**Authors:** Víctor Manuel Torres-García, Emmanuel Rodríguez-Nava, Rosa Itzel Alcántara-Rivas, Ofir Picazo, Gabriel Roldán-Roldán, Jean-Pascal Morin

**Affiliations:** 1 Department of Physiology, Faculty of Medicine, National Autonomous University of Mexico (UNAM), Mexico City, Mexico; 2 Section of Postgraduate Studies and Research, Higher School of Medicine, National Polytechnic Institute, Mexico City, Mexico

**Keywords:** Acetylcholine, Basolateral amygdala, Taste, Muscarinic receptors, Learning, Valence

## Abstract

**Rationale:**

Muscarinic receptor activity in the basolateral amygdala (BLA) is known to be involved in plasticity mechanisms that underlie emotional learning. The BLA is involved in the Attenuation of Neophobia, an incidental taste learning task in which a novel taste becomes familiar and recognized as safe.

**Objective:**

Here we assessed the role of muscarinic receptor activity in the BLA in incidental taste learning.

**Methods:**

Young adult male Wistar rats were bilaterally implanted with cannulas aimed at BLA. After recovery, rats were randomly assigned to either vehicle or muscarinic antagonist group, for each experiment. We tested the effect of specific and non-specific muscarinic antagonists administered either 1) 20 min before novel taste presentation; 2) immediately after novel taste presentation; 3) immediately after retrieval (the second taste presentation on Day 5 -S2-) or immediately after the fifth taste presentation on Day 8 (S5).

**Results:**

Non-specific muscarinic receptor antagonist scopolamine infused prior to novel taste, while not affecting novel taste preference, abolished AN, i.e., the increased preference observed in control animals on the second presentation. When administered after taste consumption, intra-BLA scopolamine not only prevented AN but caused a steep decrease in the taste preference on the second presentation. This scopolamine-induced taste avoidance was not dependent on taste novelty, nor did it generalize to another novel taste. Targeting putative postsynaptic muscarinic receptors with specific M1 or M3 antagonists appeared to produce a partial taste avoidance, while M2 antagonism had no effect.

**Conclusion:**

These data suggest that if a salient gustatory experience is followed by muscarinic receptors antagonism in the BLA, it will be strongly and persistently avoided in the future. The study also shows that scopolamine is not just an amnesic drug, and its cognitive effects may be highly dependent on the task and the structure involved.

## Introduction

The ability to recognize and overcome the value of incentives is necessary for animal´s survival, allowing them to respond quickly and appropriately to new experiences by comparing them with previous ones. Habituation is a behavioral phenomenon that is highly preserved throughout the animal kingdom and that has consistently been reported to be impaired in several neuropsychiatric disorders (McDiarmid et al. 2017). Encountering a novel taste represents a salient experience which usually manifests itself behaviorally by the phenomenon of neophobia, the tendency to hesitate and consume relatively little amounts of a new tastant, even when innately appetitive (Lin et al. 2018; Osorio-Gómez et al. 2018). On subsequent encounters, if the substance is harmless, the animal will increase its consumption, that is, present attenuation of neophobia (AN) (Bermúdez-Rattoni 2004). This incidental taste learning (Miranda et al. 2008) has been interpreted as a habituation of the anxiogenic experience of encountering a previously unknown taste (Rozin and Kalat 1971; Cintado et al. 2023).

The basolateral amygdala, (BLA) is a subcortical structure involved in emotional learning that is known to play a role in novel taste learning including AN (Gómez-Chacón et al. 2012; Shinohara and Yasoshima 2019). Inactivation of this region with GABA receptor agonist muscimol was shown to specifically eliminate palatability-related neuronal activity in the gustatory cortex (Piette et al. 2012). Meanwhile, the BLA receives the densest cholinergic projection of all forebrain structures and has high immunoreactivity to distinct types of muscarinic receptors (McDonald and Mascagni 2010, 2011). Cholinergic neuromodulation of the amygdala has been shown to play a crucial role in saliency detection and emotional learning (Aitta-aho et al. 2018; Kellis et al. 2020) mostly through muscarinic receptors activity (Crouse et al. 2020).

Earlier studies have assessed the role of muscarinic receptors, including M1, M2 and more recently M3 receptors on BLA function. The role of M1-type receptors on BLA principal neurons was recently shown to depend on the activity state of these neurons, altering their excitability in a positive feedback fashion, possibly facilitating saliency detection and emotional learning (Unal et al. 2015). M2 muscarinic receptors are also present in the BLA, probably at presynaptic sites acting as autoreceptors and providing negative feedback of acetylcholine (Ach) release (McDonald and Mascagni 2011). Finally, postsynaptic M3 receptors in this region have recently been shown to participate in balancing the relative weight of thalamic versus cortical inputs to the BLA, possibly facilitating attention to novel sensory inputs and memory encoding (Tryon et al. 2022). In the present study, we used a free choice water/taste design and assessed the function of BLA muscarinic receptors in the learning-dependent increase in taste preference that occurs in AN.

## Methods

### Animals

Adult male Wistar rats (250–300 g) were used as experimental subjects. They were housed in our vivarium in groups of five in polycarbonate cages, under a 12 h light/dark cycle, at room temperature (24ºC) with rat chow and tap water ad libitum, except where was necessary for the experiment behavioral procedures until the beginning of the experiments. All animal care and experimental procedures were approved by the Ethics Committee of the Faculty of Medicine at *Universidad Nacional Autónoma de México* (CICUAL 010–2019 / 003-CIC-2019), according to Mexican Laws for Animal Care and complied with NIH Guide for the Care and Use of Laboratory Animals. Efforts were taken to minimize the number of animals and their suffering throughout all experimental procedures.

### Surgery

Rats were anesthetized with a mixture of ketamine (100 mg/kg, i.p.) and xylazine (6 mg/kg, i.p.) administered intraperitoneally. They were next secured to a stereotaxic apparatus (Stoeling Co.) and administered the non-steroidal anti-inflammatory drug meloxicam (2 mg/kg, s.c.) and topical anesthetic (Lidocaine / Epinephrine 2%, 0.3 mL, s.c.) to ease pain. Then, the skull was exposed and cannulas made from hypodermic needles (23G) as described previously (Kokare et al. 2011) were implanted bilaterally at 1 mm above the BLA (AP: -2.8 mm; L: ± 5 mm, DV: -7.5 mm) according to rat brain atlas (Paxinos and Watson 2007) and based on previous studies (Shinohara and Yasoshima 2019; Morin et al. 2021). Afterward a stylet was placed into the guide cannula to maintain patency. Cannulas were then fixed to the skull with dental acrylic secured with a screw. Finally, rats were housed individually and allowed to recover for at least 7 days.

### Behavioral procedures

**Fig. 1:**
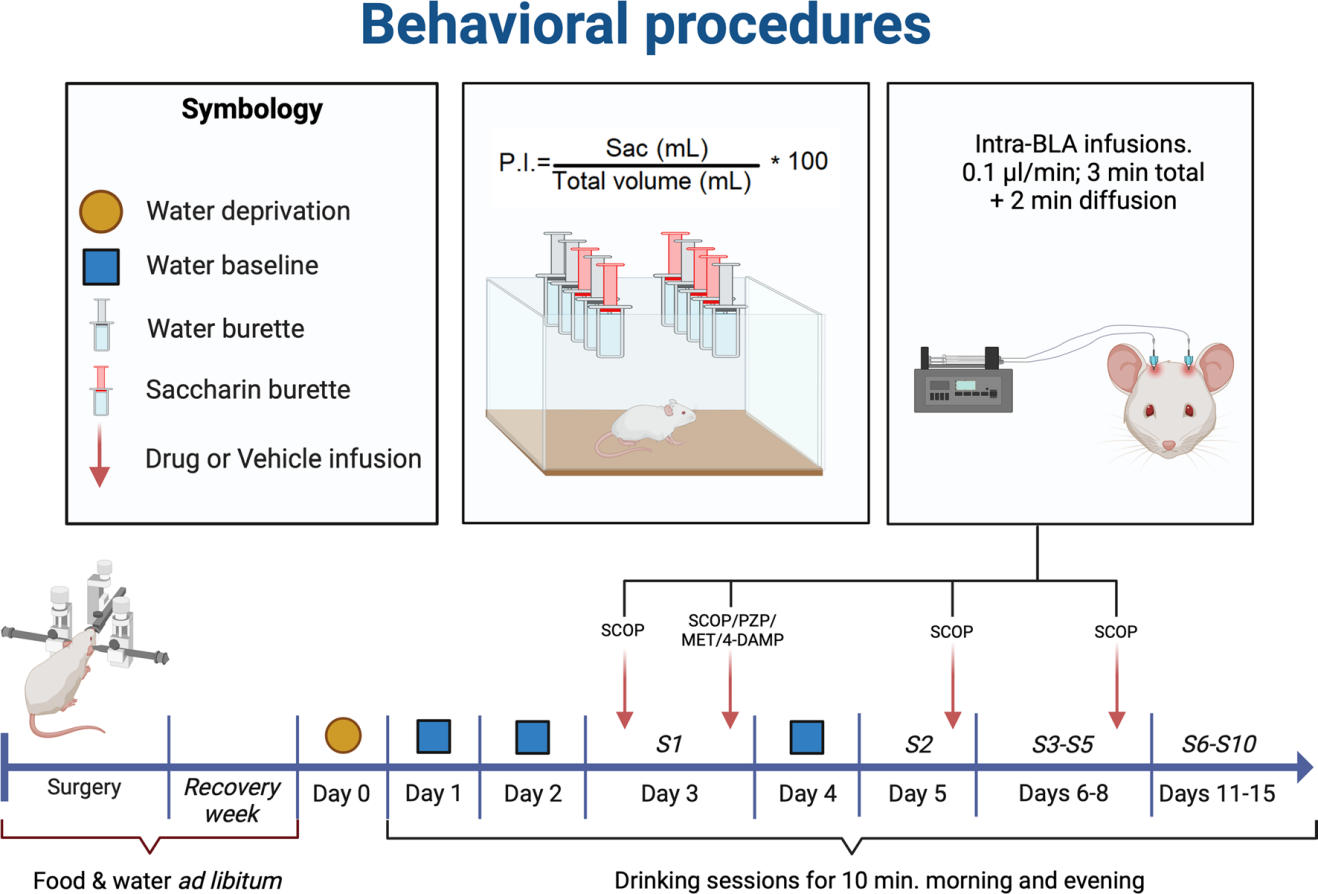
Behavioral procedures employed in the experiments described in this manuscript, unless otherwise specified

After recovery, rats were randomly assigned to either vehicle (VEH) or drug group, for each experiment. On Day 0, rats were water-deprived overnight and on the next day (Day 1) at 10:00 am they were placed in the training chamber, located in a separate room, and consisting of a polycarbonate cage (53 × 43 × 20 cm) with 10 burettes of 3 mL (5 drinking burettes at each side of the box). All rats were allowed to drink water for 10 min. This drinking season was repeated at 4 pm. This afternoon drinking session remained the same throughout the duration of the experiment. On Day 2 the procedures were identical to Day 1. These first drinking exposures were for acclimate rats to schedule and habituate them to the context in which the conditioning and testing will be taking place. On the morning of Day 3 (Neophobia, S1) 5 drinking burettes of water were replaced with a 0.3% (w./vol.) sodium saccharin solution (Sigma-Aldrich Co.) in a semi-random fashion. The procedure on Day 4 was identical to that of the first two days, consisting of only plain water drinking session. On Days 5–8 (AN S2-S5) in the morning, animals were placed in the training chamber with 5 burettes containing water and the remaining 5 containing saccharin to evaluate AN. The saccharin preference index (PI) was obtained with the following formula: vol. saccharin/ (vol. water + saccharin)*100. Behavioral and pharmacological procedures for all experiments are presented in Fig. 1.

### Drugs

The muscarinic receptor antagonist scopolamine hydrobromide (SCOP; Sigma-Aldrich, S1875) was dissolved in physiological saline (30 µg/µL) based on a pilot study as well as a previous report from our group(Morin et al. 2021). This concentration is slightly lower than the ones previously used in the insular cortex in earlier incidental taste learning studies (Gutiérrez et al. 2003b; Ramírez-Lugo et al. 2003). M1 receptor antagonist pirenzepine (PZP; Sigma-Aldrich) was used at a concentration of 45 µg/µL and M2 receptor antagonist methoctramine tetrahydrochloride (MET; ChemCruz) was used at concentration of 30 µg/µL based on previous studies (Power et al. 2003; Ramírez-Lugo et al. 2003). The concentration of M3 receptor antagonist 4-DAMP methiodide (Sigma-Aldrich) was 30 µg/µL, based on unpublished observations from our lab in which it prevented conditioned taste aversion learning (CTA) in rats when infused in the BLA before novel taste intake (data not shown). Solutions were prepared freshly just before administration or stored at -20 ºC and slowly thawed before infusion.

### Brain microinfusions

Rats were gently restrained, and the injector (30G) was inserted through the guiding cannulae of each hemisphere protruding 1 mm beyond the cannulae. A polyethylene tube attached to a 10-µL Hamilton syringe driven by automated slow-rate microinfusion pump was connected the injector. A total volume of 0.3µL/hemisphere of drug or vehicle was delivered in BLA at rate of 0.1 µL/min and the injector remained inserted for two additional minutes to allow diffusion away from the injector tip. Depending on the experiment, intra-BLA SCOP infusions were performed either 20 min before novel taste (S1) presentation or immediately after S1, S2 or S5. For specific M1, M2 or M3 receptor antagonism experiments, infusions were after S1. 

### Histology

Once the behavioral procedure was concluded, rats were administrated an overdose of sodium pentobarbital (100 mg/mL i.p.) and intracardially perfused with PB 0.1 M (4ºC) followed by paraformaldehyde (PFA; 4% w./vol. in PB). Brains were post-fixed overnight in PFA and were transferred on the next day to a cryoprotective solution consisting of sucrose 30% (w./vol.) and sodium azide 0.02% (w./vol.) in PBS 0.1 M and stored at 4ºC. Using a cryostat (Leica), coronal sections of 40 µm were obtained for Nissl staining at the site of infusion. An experimenter blind to treatment group visualized injector tip placements with an Olympus stereoscopic microscope equipped with a 4X objective lens. Subjects were excluded when at least one injector tip was outside the structure of interest.

### Statistical analysis

PIs throughout experimental days and between drugs and vehicle groups were compared using Repeated Measures 2-way ANOVAs, with Geisser-Greenhouse correction of p values using factors “Day” and “Treatment” and post-hoc Tukey tests were performed where indicated. In all cases, statistical significance was set at *p* < 0.05. All analyses and graphs were performed using Prism10 (GraphPad Software, San Diego, CA, USA). Each experiment was performed at least twice, totaling between 7 and 11 animals per group.

## Results

### Scopolamine infusion in BLA before the novel taste prevents AN

**Fig. 2:**
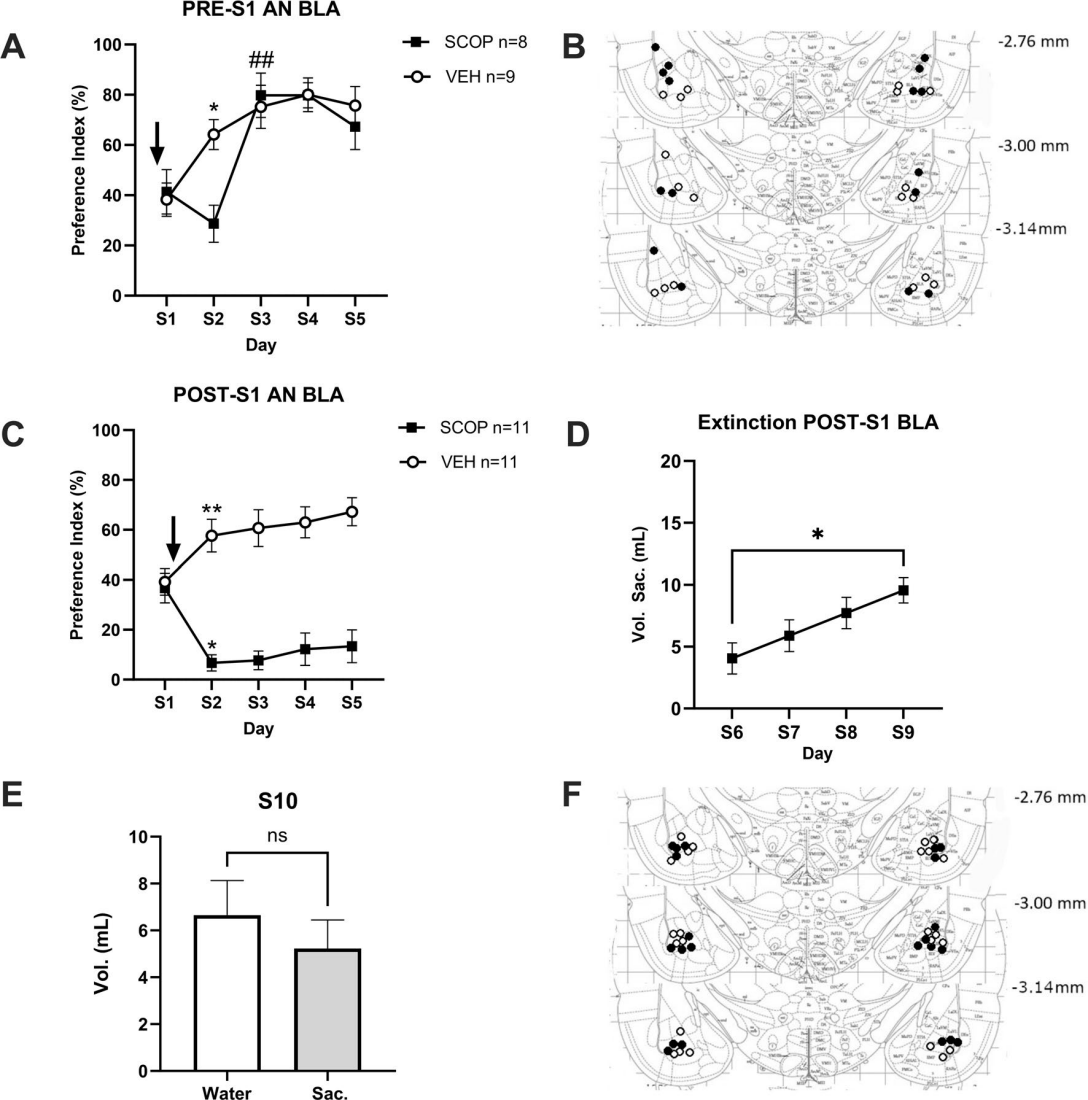
**A** Effect of bilateral intra-BLA SCOP infusion 20 min before novel taste exposure on AN; *: *p* < 0.05 vs. S1 of same group; ##: *p* < 0.01 vs. S2 of same group. **B** Injector tips placements for rats included in A). **C** Bilateral intra-BLA SCOP infusion immediately after novel taste intake (S1) produced a marked decrease in taste preference on S2; **: *p* < 0.01 vs. S1 of same group. **D** Saccharin intake in mL in rats of the SCOP group of the experiment shown in C) from days 6 to 9 where they were exposed to saccharin only in the morning session; *: *p* < 0.05. **E** Fluid intake in mL of these same rats on day 10, where they had the choice between saccharin and water. ns: not statistically significant. **F** Injector tips placements for rats included in C)

To test the role of muscarinic receptors in the BLA in the acquisition of AN, we performed intra-BLA SCOP or VEH infusions 20 min before novel taste presentation (S1). First, both the SCOP and VEH groups consumed similar amounts of fluid (water + saccharin) across the duration of the sessions, no main effect of Treatment (F_(1,15)_ = 1.04, *p* > 0.32 or Treatment x Day interaction (F _(4,60)_ = 2.12, *p* = 0.09) being observed (data not shown). This observation suggests that under our conditions, SCOP did not significantly affect thirst or motivation to drink. As for saccharin preference (PI, see methods), a significant interaction between Day and Group variables (F_(4, 60)_ = 3.59, *p* < 0.05) was observed. On novel taste presentation day, SCOP and VEH groups consumed similar amounts of saccharine solution (PIs = 38.28 ± 6.6% vs. 41.38 ± 8.8% for VEH and SCOP, respectively, *p* > 0.78) suggesting that intra-BLA scopolamine infusions had no effect on taste perception or neophobic reaction (Fig. 2A-B). Tukey post hoc test showed that there were significant intragroup differences between S1 and S2 in the VEH group (PI´s = 38.28 ± 6.6% and 64.17 ± 6.0% for S1 and S2, respectively, *p* < 0.05) consistent with the phenomenon of AN. In the SCOP group however, no difference was observed between S1 and S2 (41.38 ± 8.8% vs. 28.67 ± 7.4% for S1 and S2, respectively, *p* > 0.73) suggesting that intra-BLA SCOP impaired the ability to form AN. In this group however AN was evident on the following day (i.e., S3) as the PI increased to 79.82 ± 8.8% compared to 28.67 ± 7.4% on the previous day (*p* < 0.05; see Fig. 2A-B). Together, these data suggest that intra-BLA SCOP infusions prior to novel taste intake prevents AN formation but does not affect the neophobia.

### Scopolamine infusion in BLA after novel taste intake produces conditioned taste avoidance

In our second experiment we assessed the effects of scopolamine infusions in the BLA immediately after novel taste intake. Here, surprisingly, when SCOP was infused immediately after novel taste consumption, animals showed a strong avoidance to the taste on the second presentation, two days after. A significant interaction was observed between the Day and Group variables (F_(4,80)_ = 21.47, *p* < 0.0001; Fig. 2C). Post hoc Tukey test unveiled a significant increase in the PI in the VEH group (PI´s = 39.21 ± 5.3% and 57.65 ± 6.6% for S1 and S2, respectively, *p* < 0.01), consistent with AN in this group. In the SCOP group, however a significant *decrease* in the PI was observed from S1 to S2 (PI´s = 36.72 ± 5.9% and 6.72 ± 3.2%, respectively, *p* < 0.05; see Fig. 2C), suggesting that muscarinic receptors inhibition in the BLA posterior to novel taste consumption promoted avoidance to that taste. Subsequent sessions with free-choice of saccharin and water on the next three days (S3-S5) showed that this marked decrease in the PI was maintained although by S5, it was no longer significantly different from S1 (*p* = 0.14) possibly highlighting the start of an extinction process see Fig. 2C).

We further submitted this SCOP-induced taste avoidance to extinction by performing the next 4 drinking sessions (S6-S9) with all drinking burettes containing the saccharin solution and giving them the choice between saccharin and water again on the following day (S10). Indeed, although rats did not present preference compared to water on this day, they did not show avoidance to saccharin either (PI = 45.13 ± 9.0), indicating they underwent extinction, although perhaps not complete by this point (Fig. 2D-F). The fact that 4 additional “forced” saccharin drinking sessions were required underscore the robustness of the taste avoidance induced by intra-BLA SCOP infusion after taste intake.

### Intra-BLA scopolamine-induced taste avoidance does not generalize to another novel taste

**Fig. 3:**
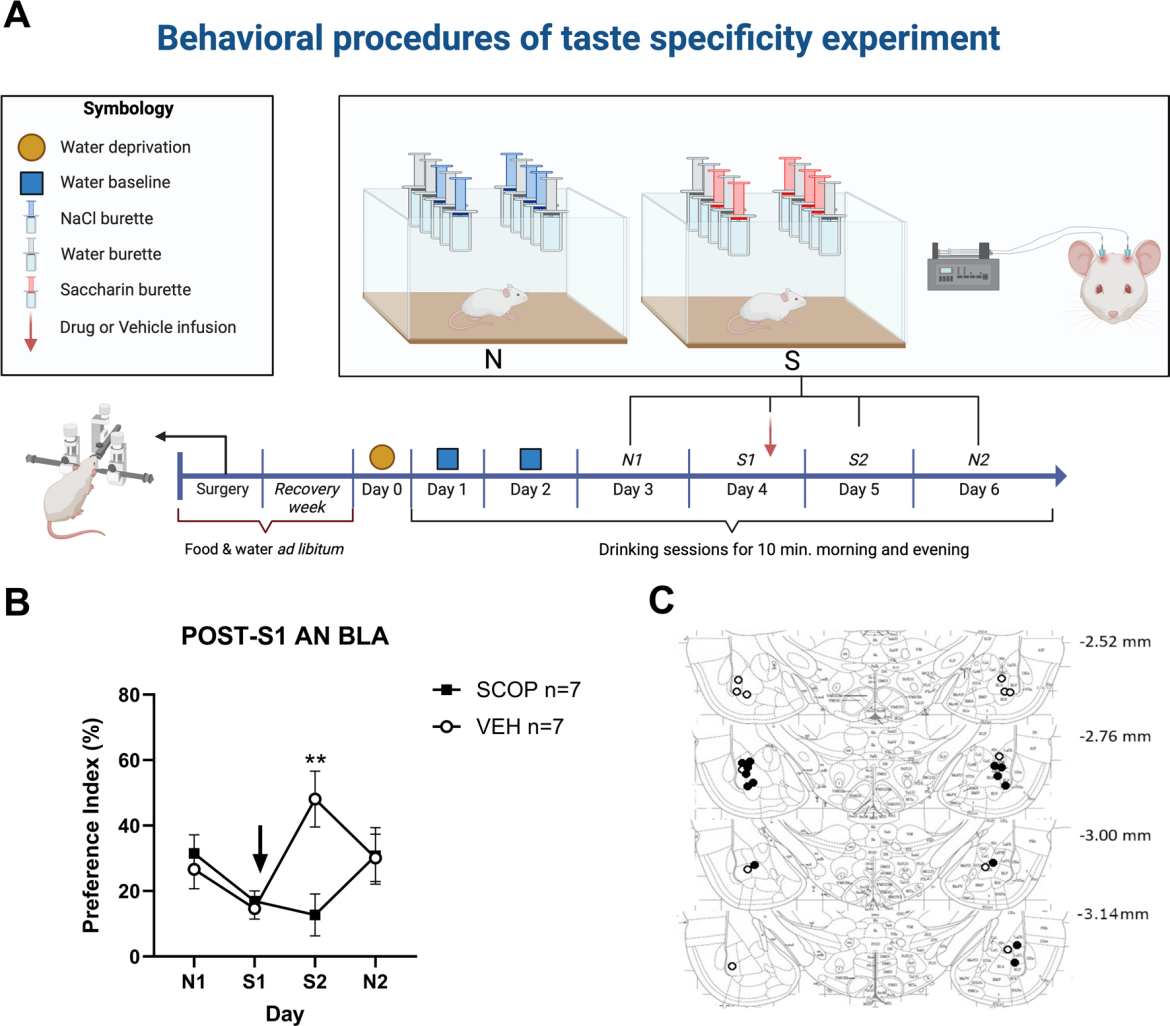
**A** Behavioral procedures for the experiment aimed at assessing the possibility of generalization of the SCOP-induced taste avoidance. **B** Taste avoidance observed on S2 after post-S1 bilateral intra-BLA SCOP infusion did not extend to another novel taste (NaCl); *: *p* < 0.05 vs. S2-VEH. **C** Injector tips for rats included in B)

**Fig. 4:**
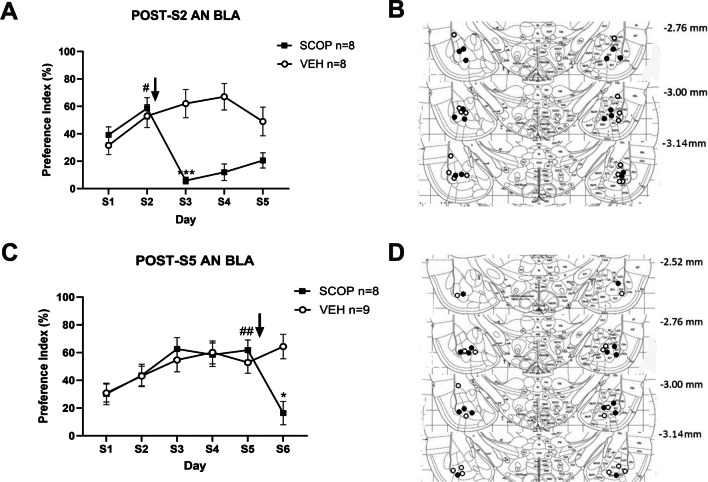
Effect of intra-BLA SCOP infusion immediately after S2 (**A**) or S5 (**C**). **B**-**D** injector tip placements for rats included in A and C, respectively

It has been demonstrated that under certain circumstances, animals conditioned to avoid saccharin initially generalize this avoidance to other novel tastants (Wu et al. 2021; Ramos et al. 2022). Given our observation that intra-BLA SCOP infusions in the BLA after novel taste consumption appeared to produce a clear conditioned taste avoidance, we next evaluated whether this pharmacologically induced avoidance was specific to the “conditioned” taste or if it could generalize to other novel tastes. For this, we presented rats with another different taste (0.15 M NaCl in tap water) that was not previously paired with intra-BLA SCOP infusions. When comparing taste preference for saccharin that was previously paired with intra-BLA SCOP infusions versus taste preference for NaCl, a significant Treatment x Taste interaction was observed (F_(3,36)_ = 4.96, *p* < 0.01, Fig. 3). The neophobic reaction to saccharin in this set of experiments was exceptionally strong with a 15.8% PI when combining VEH and SCOP groups. This is possibly why we did not observe a significant drop in the PI on the second presentation in the SCOP group (12.7% on S2, *p* > 0.91). The VEH group on the other hand did present a significant AN on S2 (from 14.66% ± 3.2% on S1 to 48.11 ± 8.5% on S2, *p* < 0.01). Given our earlier observations presented on Fig. 2, as well as those from subsequent experiments (see Fig. 4) we attribute the lack of increase in the PI to a taste-avoidance inducing rather than an amnesic effect by SCOP.

When comparing the PI for the second NaCl presentation (N2), no difference was observed between groups (PI N2 = 30.11 ± 7.2% vs. 30.73 ± 8.7%, for VEH and SCOP, respectively, Sidak post hoc test *p* > 0.72; Fig. 3). This suggests that post-taste intra-BLA SCOP infusions produces a conditioned taste avoidance that is specific and does not generalize to other novel tastes.

### Saccharin pre-exposition for several days does not prevent intra-BLA scopolamine-induced taste avoidance

We assessed whether this taste avoidance-producing effect was dependent upon the novelty of the taste stimulus. Under a one-bottle experimental paradigm, in which rats are presented with saccharin only without the option to drink plain water, AN has been described as a graded learning task in which animals usually reach asymptotic performance by the 4th or 5th taste presentation (Rodriguez-Ortiz et al. 2005; Morin et al. 2011). We therefore performed two additional experiments, the first with intra-BLA SCOP infusions after AN retrieval (post-S2) and the second with the same treatment administered after familiar taste intake (post-S5). In the first experiment, the data obtained showed a significant interaction between Day and Treatment (F_(4, 56)_ = 23.44, *p* < 0.0001). Post hoc Tukey test detected a marked decrease in the PI in the SCOP group from S2 to S3 (PI = 58.60 ± 7.7% and 5.89 ± 2.4% SCOP *p* < 0.001; Fig. 4A-B), suggesting that muscarinic receptors inhibition in the BLA after AN retrieval promoted avoidance to the taste on the third presentation. In the second experiment, the data yielded a significant interaction between Day and Group (F_(5, 75)_ = 7.374, *p* < 0.0001). Post hoc Tukey test showed a pronounced decrease in PI in SCOP group from S5 to S6 (PIs = 61.83 ± 7.3 vs. 16.35 ± 8.4%, *p* < 0.05; Fig. 4C-D). Therefore, although a weak latent inhibition (LI) appears to have occurred in the post-S5 experiment (the PI of S6 was comparable to that of S1, *p* > 0.89) our data show that even when rats were previously familiarized with the taste, infusions of SCOP in the BLA after its consumption produced conditioned taste avoidance.

### M1/M3, but not M2 antagonism in BLA following novel taste produces a moderate avoidance to the taste on subsequent encounters

**Fig. 5:**
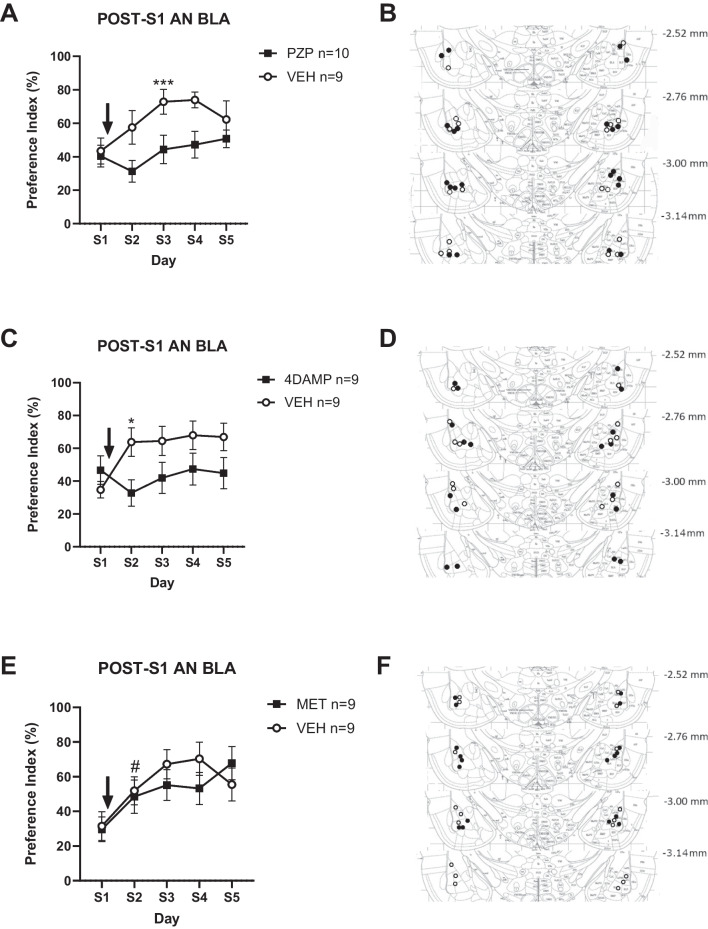
Effect of bilateral intra-BLA PZP (**A**-**B**), 4-DAMP (**C**-**D**) and MET (**E**–**F**) immediately after novel saccharin intake on AN. *: *p* < 0.05 vs. S1 of same group; ***: *p* < 0.001 vs. S1 of same group; #: *p* < 0.05 MET + VEH S1 vs. S2

We next assessed the effect of administering specific muscarinic receptors antagonists in the BLA after novel taste intake. First, M1-type receptor antagonist PZP was bilaterally infused in the BLA immediately after novel taste intake and subsequent taste intake was compared to their VEH counterparts. A significant interaction between Treatment and Day variables F_(5, 90)_ = 2.54, *p* < 0.05) was observed. Post hoc analysis unveiled that VEH animals did not reach a significant increase in their PI by S2 but did so by S3 (PI´s = 43.47 ± 7.7% and 72.86 ± 7.4% for S1 and S3, respectively, *p* < 0.001; Fig. 5A-B). This increase, however, was not observed in the PZP group: S1 and S3 PIs = 40.41 ± 6.6% and 44.42 ± 8.5%, respectively, *p* > 0.99, indicating that AN did not occur in these rats (see Fig. 5A-B).

Then we evaluated the effect of bilateral, intra-BLA infusion of M3 receptor antagonist 4-DAMP after novel taste. Here again, we observed a significant interaction between Day and Treatment variables F_(4, 64)_ = 4.63, *p* < 0.01). Tukey post hoc tests showed that VEH had a significant IP increase from S1 to S2 (PI’s = 34.74 ± 5.0% and 63.76 ± 8.7%, respectively (n = 9), *p* < 0.05; Fig. 5 C-D). This increase was not observed in the 4-DAMP-infused group (PIs = 46.71% ± 8.7% vs. 32.77 ± 8.1, for S1 and S2, respectively, *p* > 0.74) again indicating that AN did not occur in this group (see Fig. 5 C-D).

Finally, we tested the effect of inhibiting presynaptic M2 receptors by infusing MET intra-BLA after S1. Data showed that there was no interaction between Day and Drug F_(4,64)_ = 1.53, *p* > 0.20. There was, however, a main effect of Day (F_(4,64)_, = 8.80, *p* < 0.0001; Fig. 5 E–F), indicating safe taste memory formation regardless of treatment.

## Discussion

The present study was aimed at determining the effect of BLA muscarinic receptors antagonism in incidental taste learning, a type of learning that entails both habituation to the initial neophobic response as well as an incremental approach behavior on subsequent presentations of the taste (Bermúdez-Rattoni 2004; Neath et al. 2010). Our experimental design implied that rats had free choice between water and saccharin both in the first taste presentation and in every subsequent tests. We show that intra-BLA SCOP infusions before novel taste presentation has no effect on the preference of the novel taste relative to water, suggesting that taste perception and its saliency as novel was not affected by this treatment, similar to what has been reported in the insular cortex (Gutiérrez et al. 2003b). Also arguing against a possible effect of SCOP on taste perception *per se* is a previous study from our group which showed that when it is infused in the BLA before the test phase in a conditioned taste aversion (CTA) paradigm, animals show a clear avoidance to the taste (Morin et al. 2021) indicating that they can perceive it.

Control rats with intra-BLA infusion of vehicle before novel taste presentation showed a significant increase in saccharin preference on the second presentation the taste, consistent with the phenomenon of AN;this increase was abolished in the SCOP group. This initially pointed to an amnesic-like effect of the drug, as was reported when infused in either the insular cortex or the perirhinal cortex, reporting impaired AN memory formation in both cases (Gutiérrez et al. 2003a, 2004). Similarly, rats with BLA lesions also show impaired AN (Gómez-Chacón et al. 2012). Our subsequent experiment, however, suggested that the lack of AN in the SCOP group could also be due to a lingering effect of the SCOP infused 20 min before taste presentation. Indeed, in our next experiment where SCOP infusions were performed after novel saccharin consumption, a robust avoidance behavior was formed for that taste as the preference index drastically dropped on the second exposure performed two days later. On the other hand, a previous study from our group showed intra-BLA SCOP infusion before novel taste consumption produced a strong amnesic effect in a CTA paradigm (Morin et al. 2021). This may suggest that our present results of AN impairment in rats infused with SCOP before novel taste intake may indeed be due to an amnesic effect of the drug rather than a lingering aversive conditioning. Also arguing against a possible lingering effect of SCOP is the fact that rats infused with the drug before the first saccharin presentation showed a robust increase in the taste’s preference from the second to the third presentation, which was not observed when SCOP was administered after S1 or S2 where extinction of the SCOP-induced taste avoidance was very slow probably due to the characteristics of our behavioral protocol (see below), nor when PZP or 4-DAMP was administered after S1 in which drug-infused groups failed to increase their preference compared to S2 on subsequent days. Therefore, the observation that SCOP infusions in the BLA after the taste produced a strong avoidance on subsequent encounters suggests that interfering with muscarinic signaling post-acquisition may have distinct effects compared to pre-taste SCOP which appears to impair AN learning, as it does in the CTA task (Morin et al. 2021). More studies are warranted to assess whether the temporality of muscarinic inhibition in the BLA may cause distinct behavioral effects.

Besides the robust learned avoidance produced by post-taste intra BLA-SCOP infusions, another observation was that it appeared relatively resistant to extinction. The fact that we observed such a slow extinction however could be attributed, at least in part, to inherent characteristics of our behavioral design, such as the saccharin concentration (0.3% in our case) and the free-choice, multiple bottles drinking sessions in a context that differs from the rats’ home cage. First, the fact that we used a 0.3% saccharin concentration, which is suited for incidental taste learning experiments but considerably higher than the 0.1% concentration usually used, especially in CTA experiments probably contributed to our suboptimal extinction. As is the case for the US, a more concentrated or salient CS has long been known to produce a stronger CTA which is slower to extinguish (Nowlis 1974; Braun and Rosenthal 1976). CTA by itself has been identified as a hard-to-extinguish aversive memory (Mickley et al. 2012) and even at a 0.125% concentration, when the tests are performed in a free choice saccharin/water setup like the one we used in here, rats start to show a -slight- preference for the taste only by day 9 (Nunnink et al. 2007). Additionally, CTA extinction was reported to be slower when the experiment is performed in an experimental cage, distinct from their home cage (De La Casa et al. 2003), as is the case in our study.

Another intriguing observation from the current data is that by submitting the rats to up to four saccharin pre-exposures we observed only very modest LI (see Fig. 4). Besides the relative robustness of the SCOP-induced taste avoidance we report in here, we believe that this weak latent inhibition is partly due to our behavioral design in which in all taste exposures, rats also have the choice to drink plain water, possibly mitigating the anterograde interference of such pre-exposures. In addition, shorter water deprivation schedules like the one used in the present manuscript -all rats had an additional water-only drinking session in the afternoon- have been reported to have an attenuating effect on LI (De la Casa and Lubow 1995). Similarly, the fact that our drinking sessions were performed in a different context may have reduced the capacity to form LI (Quintero et al. 2011).

Earlier studies have assessed the effect of antagonizing different neurotransmitter systems at the BLA on incidental taste learning, none of which, to our knowledge, reported an avoidance-inducing effect as the one we report herein. Several of these works however did report impaired long-term taste habituation (i.e. AN). For instance, β-adrenergic receptor antagonist propranolol infused in the BLA 5 min before novel taste intake (a highly preferred, diluted saccharin solution with low neophobic properties in this case) impaired taste habituation when evaluated 3 days later (Miranda et al. 2008). Similarly, NMDA receptor antagonism in the BLA prior to the presentation of a novel 0.5% saccharin solution prevented AN (Figueroa-Guzmán and Reilly 2008). More recently, the role of D1 receptors of the amygdala, which is known to be densely innervated by dopaminergic projections (McDonald 2020) where assessed and it was shown that infusion of antagonist SCH23390 *after* 3% cider vinegar intake was without effect, while the agonist SKF-81297 impaired AN. Finally, temporal inactivation of the BLA with the GABA antagonist cocktail baclofen/muscimol after novel taste consumption had little effect on AN, although interestingly, the same treatment performed before novel taste intake abolished the expression of neophobia (Lin et al. 2018).

How could SCOP infusions in the BLA produce such a marked conditioned taste avoidance? Using a different behavioral setup, a recent study also reported a pharmacologically-induced taste avoidance by following novel taste exposure with concomitant activation of glutamatergic and adrenergic receptors in the amygdala (Osorio-Gómez et al. 2019). This work, in contrast with the data presented here, was based on previous observations of increased release of glutamate and noradrenaline in the amygdala after intraperitoneal injection of the gastric malaise-inducing agent LiCl (Guzmán-Ramos et al. 2012); mimicking the neurochemical events in the amygdala produced by the irritating agent partly induced the subsequent taste avoidance behavior. In the present case however, ACh release in the BLA is not known to signal malaise or suppression thereof but has been reported to promote the learning of approach behavior (Aitta-aho et al. 2018). This same study also showed that BLA-projecting Nucleus Basalis Magnocellularis neurons are specifically activated by an appetitive stimulus but not by an aversive one. In addition, to the degree that we may assume that intra-BLA SCOP induced some form of negative experience, it has been showed that in situations such as high levels of anxiety, normally rewarding stimuli can acquire a negative valence, through the activation of “aversive” networks in the amygdala during a specific experience (Pignatelli and Beyeler 2019). It is therefore possible that our blocking of muscarinic signaling in the BLA, disrupted valence-assigning networks in the BLA, favoring the activation of negative valence neurons upon saccharin reinstatement. The fact that our SCOP-induced avoidance did not seem to generalize to another taste may support this possibility.

It was recently demonstrated that distinct populations of BLA principal neurons encode stimuli of opposing valence (Kim et al. 2016). In this regard, while some authors have shown evidence that neurons encoding positive and negative valence are morphologically, spatially and genetically segregated (Kim et al. 2016), other data suggest that the valence assigned to BLA neurons are more dependent on their specific efferent routes (Zhang et al. 2021). In addition, it was shown that valence assignment in BLA neurons is highly plastic and may be regulated by neuromodulatory inputs (Li et al. 2022). Interestingly, recent data shows that muscarinic signaling differentially shifts neuronal activity in distinct BLA interneuron subpopulations, effectively inducing a plasticity-prone state at pyramidal cells post-synaptic sites by reducing GABAergic input at distal dendritic sites (Bratsch-Prince et al. 2023). Both these effects are suggestive of muscarinic-dependent plasticity mechanisms underlying emotional memory in this region and given the role of BLA ACh signaling in valence assignment (Aitta-aho et al. 2018), it is possible that our interfering with muscarinic signaling after gustatory experience disturbed its stored valence component. Besides the Nucleus Basalis Magnocellularis which for the bulk of the cholinergic projections to the BLA (Aitta-aho et al. 2018), the Ventral Pallidum also contains a small population of cholinergic neurons that project to the BLA, among other structures (Root et al. 2015). This small population has recently been shown to contain neurons that encode the valence of olfactory stimuli and chemogenetic inactivation of those cholinergic Ventral Pallidum neurons activated by odors of positive valence, switched the odor’s valence to aversive (Kim et al. 2024). Further studies should examine the specific cholinergic circuit involved in the acquired taste avoidance described in here. We also showed that specific M1/M3 antagonism in the BLA after novel taste exposure prevented AN but failed to produce an avoidance as strong as that produced by SCOP infusions. Similar to our result in the pre-S1 SCOP experiment (see Fig. 2A) two mutually exclusive interpretations could explain these observations, that is, an amnesic effect of both PZP and 4-DAMP on AN memory formation or a weak conditioned avoidance produced by these interventions. We believe that the latter seems more likely. Besides the fact that the drug infusions were performed after taste intake, another striking difference with our pre-S1 results is that on subsequent taste presentations (i.e. S3-S5) the PIs remain similar to those of S2 as is the case with the post-taste SCOP infusion experiments, that is, no “extinction” is observed. This contrasts with the pre-S1 results in which a marked increase in the PI is observed in the SCOP group from S2 to S3.

Noteworthy, although 4-DAMP is mainly a M3-receptor antagonist, it has some affinity for the M1 receptor (Jeon et al. 2013). It is thus possible that our 4-DAMP results, which are very similar to those obtained with pirenzepine, may be due to M1 receptor antagonism, especially considering the fact that M1 receptors are highly abundant in the BLA (McDonald and Mascagni 2010). Muscarinic stimulation has previously been shown to produce hyperpolarization of BLA principal neurons through small conductance calcium-activated (SK) potassium channel activation (Power and Sah 2008). ACh-in release in the BLA was also shown to produce M1-dependent IPSPs in quiescent, but not in active pyramidal neurons, enhancing the signal to noise ratio, which could serve as a mechanism to facilitate the initial acquisition of associative memory (Unal et al. 2015). More recently, ACh was reported to affect BLA activity by strongly inhibiting glutamate release from prelimbic cortical projections -which are known to be involved in emotional learning (Diehl et al. 2020)- through M4-mediated presynaptic inhibition (Tryon et al. 2022). To a lesser extent medial thalamic nucleus input to BLA is inhibited through M3-dependent stimulation of retrograde endocannabinoid release (Tryon et al. 2022). Therefore, precise contribution of specific muscarinic receptor pathways is probably required to assign an appropriate valence to previously encountered experiences. Overall, examining role of muscarinic signaling in the BLA, both at the cellular and behavioral level represents a promising area of study to deepen our understanding of emotional learning in both normal and pathological conditions.
